# Case report: A case of severe retropharyngeal edema after COVID-19 successfully treated with intravenous immunoglobulin

**DOI:** 10.3389/fped.2023.1198505

**Published:** 2023-07-18

**Authors:** Takanori Suzuki, Toya Kono, Hisada Satoshi, Hidetoshi Uchida, Seiichiro Ota, Ichiro Tateya, Tetsushi Yoshikawa

**Affiliations:** ^1^ Department of Pediatrics, School of Medicine, Fujita Health University, Aichi, Japan; ^2^ Department of Otorhinolaryngology, School of Medicine, Fujita Health University, Aichi, Japan; ^3^ Department of Radiology, School of Medicine, Fujita Health University, Aichi, Japan

**Keywords:** COVID-19, retropharyngeal edema, MIS-C, IVIG (Intravenous immunoglobulin) administration, kawasaki disease (KD)

## Abstract

Multisystem inflammatory syndrome in children (MIS-C) has been widely reported, mainly in Western countries. The clinical features of MIS-C and Kawasaki disease are similar. The latter is common in Asian countries, including Japan. Meanwhile, the incidence of MIS-C seems to be low in Japan. Retropharyngeal edema is relatively common in older patients with Kawasaki disease and has been reported in a few patients with MIS-C. We describe a case of severe retropharyngeal edema after coronavirus disease 2019 (COVID-19) that improved quickly with high-dose of intravenous immunoglobulin treatment. Onset of retropharyngeal edema was 3 weeks after COVID-19. The patient received appropriate intravenous antibiotics for 5 days, but his symptoms worsened. Therefore, we suspected that his retropharyngeal edema was caused by suspected MIS-C even though he did not have the typical clinical symptoms of suspected MIS-C such as gastrointestinal symptoms and shock. Retropharyngeal edema was refractory to antibiotic therapy but lessened quickly with high-dose immunoglobulin therapy, without other typical clinical manifestations of MIS-C, suggesting that early immunoglobulin therapy might prevent the progression of MIS-C.

## Background

Although coronavirus disease 2019 (COVID-19) is generally a mild disease in children, severe inflammation of multiple organ systems, a condition termed multisystem inflammatory syndrome in children (MIS-C), has been reported, mainly in Western countries (1, 2). The American College of Rheumatology has published a diagnostic algorithm for MIS-C that includes clinical features (rash, gastrointestinal symptoms, edema of the hands or feet, oral mucosal changes, conjunctivitis, lymphadenopathy, and neurologic symptoms), as well as a tiered laboratory workup (3–5). Specific treatments such as intravenous immunoglobulin and steroids are required to improve the prognosis of MIS-C. Since MIS-C and Kawasaki Disease share similar clinical features, a detailed differential diagnosis of the two diseases; this is especially necessary as Kawasaki Disease is common in Asian countries, including Japan (6, 7). Retropharyngeal edema is a characteristic finding that is relatively common in older patients with Kawasaki disease (8). Meanwhile, retropharyngeal edema has also been demonstrated in a small number of patients with MIS-C. Here we report a case of severe retropharyngeal edema that was refractory to antibiotic therapy but rapidly resolved with high-dose immunoglobulin therapy. The patient had been infected with COVID-19 three weeks before the onset of the disease and had recovered without any other clinical manifestations of MIS-C.

## Case presentation

**Figure 1:**
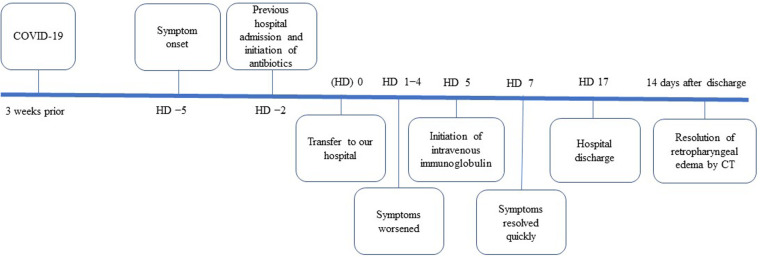
Timeline of key events, HD; hospital days.

A previously healthy nine-year-old Japanese boy was transferred from another hospital for refractory severe retropharyngeal edema despite intensive intravenous antibiotic therapy for two days. The patient's clinical course is shown in Figure 1. He had COVID-19 three weeks prior to the onset of this disease. The antigen test for COVID-19 was positive. At that time, he had a high fever for 5 days but recovered without any complications or sequelae. Five days before admission to our hospital, he had a high fever of 39°C. Rapid antigen tests for influenza and SARS-CoV-2 performed in the local pediatric clinic were negative. Subsequently, in addition to high fever, other symptoms such as headache, neck tenderness, limitation of neck motion, muffled voice, difficulty swallowing, and severe snoring developed. Two days before admission to our hospital, he was admitted to a local hospital due to a severe inflammatory response; white blood cell count (WBC) was 25,200/μl and C-reactive protein (CRP) was 31 mg/dl. Contrast-enhanced CT(computed tomography) showed posterior pharyngeal edema. Since posterior pharyngeal abscess was suspected, ceftriaxone (1000 mg four times daily), clindamycin (300 mg four times daily), and vancomycin (500 mg four times daily) were administered. These antibiotics of the treatment started at the local hospital where the patient was first admitted. On the second day of admission to the local hospital, the patient was transferred to our hospital for surgical treatment.

On admission to our hospital, he had high fever, pharyngeal erythema, and bilateral cervical lymphadenopathy with tenderness. However, he did not have any typical symptoms of Kawasaki disease, such as ocular conjunctival hyperemia, lip redness, fingertip edema, or skin rash. He was nine-years old, which was not the preferred age for Kawasaki disease. In addition, he had no gastrointestinal symptoms. His abdomen was soft, non-tender, and non-distended. A repeat rapid antigen test for SARS-CoV-2 performed on admission to our hospital was negative. No clinical symptoms suggestive of heart failure were observed at hospital admission.

**Figure 2:**
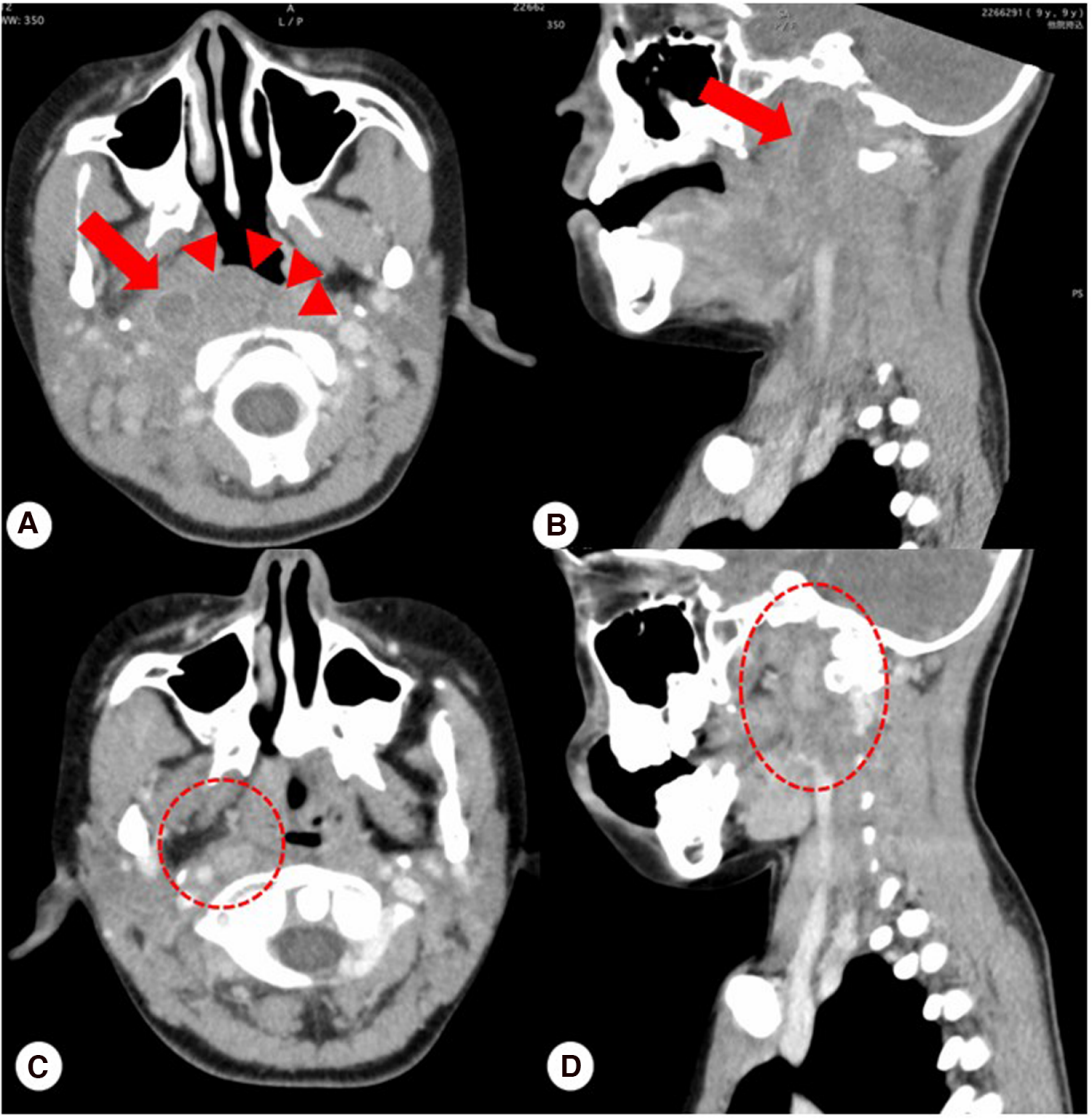
(**A,B**) Contrast-enhanced CT showed retropharyngeal edema on the right side in an area of 17 mm × 14 mm × 34 mm indicated by arrows, edema appearing as a hypo-absorptive area in front of the vertebral body on C1 indicated by arrowheads, and bilateral tonsillar enlargement. (**C,D**) Follow-up contrast-enhanced CT scan at 14 days after discharge confirmed complete resolution of retropharyngeal edema and no sign of edema indicated by ovals.

His initial workup was significant for leukocytosis (WBC count, 26,400/μl; absolute neutrophil count, 22,704/μl; absolute lymphocyte count, 1,150/μl), elevated CRP (23 mg/L) and erythrocyte sedimentation rate (104 mm/hour), and decreased sodium level (129 mEq/L). N-terminal pro-brain natriuretic peptide and Troponin I were normal (74 pg/ml, 0.0012 ng/ml). Furthermore, fibrinogen and D-dimer were elevated (881 mg/dl, 3.8 μg/ml). The patient's electrocardiogram was unremarkable, showing good cardiac contraction, no pericardial effusion, and no coronary abnormalities. This case was not vaccinated against COVID-19, and serological evaluation of COVID-19 was not been performed. Fiberoptic examination of the larynx revealed severe swelling of the palatine tonsils, mild swelling of the epiglottis, and salivary effusion. Contrast-enhanced CT showed right posterior pharyngeal edema in an area of 17 mm × 14 mm × 34 mm, a hypo-absorptive area in front of the vertebral body on C1 suggestive of edema, and bilateral tonsillar enlargement (Figures 2A,B). Although no bacteria were isolated from a throat swab or blood cultures performed at the first visit to the local hospital, the three antibiotics had been given continuously after transfer to our hospital. The patient received the three antibiotics for a total of sevens days, but his symptoms worsened.

Due to a poor response to the three antibiotics and the previous history of COVID-19, and no bacteria were isolated from a throat swab or blood cultures performed at the local hospital and our hospital, we suspected early-stage MIS-C. In addition, it was difficult to perform surgical treatment. The patient was at high risk for upper airway obstruction due to rapidly progressive retropharyngeal edema. Thus, we diagnosed the patient with suspected MIS-C on the fifth day of admission and administered single 10% intravenous immunoglobulin (2 g/kg). Thereafter, his symptoms rapidly lessened. The patient was discharged on the 17th day of hospitalization with no findings of myocardial damage or coronary artery dilatation based on echocardiography in addition, CRP 0.13 mg/L confirmed negative. A follow-up contrast-enhanced CT scan 14 days after discharge showed complete resolution of retropharyngeal and posterior pharyngeal edema (Figures 2C,D). Consent for publication was obtained from patient and his guardians according to the institution's guidelines.

## Discussion

In the present case, a previously healthy nine-year-old boy developed severe retropharyngeal edema refractory to intensive antibiotic treatment. He had the typical clinical signs and symptoms of retropharyngeal edema and enhanced CT showed the typical findings of the disease. In addition to the lack of response to antimicrobial treatment for severe retropharyngeal edema, the throat swab and blood cultures performed at the first hospital visit were negative. Furthermore, he had COVID-19 three weeks prior to the onset of retropharyngeal edema. He was nine-years old, which the preferred age for MIS-C (7, 9).

There have been several case reports of MIS-C complicated by retropharyngeal edema (9–14). In one study, neck-related symptoms occurred in 39 of 137 (28.5%) patients with MIS-C; 4 of 137 (2.5%) patients with MIS-C had retropharyngeal edema (14). Since this patient did not have symptoms typical of Kawasaki disease such as skin rash, inflammation of the oral mucosa, conjunctival injection, extremity findings, gastrointestinal symptoms, and cardiac failure, the diagnosis was suspected to be early-stage retropharyngeal edema associated with MIS-C.

As a result of intravenous immunoglobulin treatment for MIS-C, his symptoms rapidly diminished and his laboratory data also rapidly returned to normal. After finishing the treatment, we monitored carefully for gastrointestinal symptoms and cardiac dysfunction, but neither gastrointestinal symptoms nor shock was observed. The previously reported cases of MIS-C with retropharyngeal edema included the typical clinical manifestations of MIS-C, including cardiac shock (10–12). In at least two patients with MIS-C, retropharyngeal edema clearly preceded the appearance of typical clinical symptoms of MIS-C, including severe gastrointestinal symptoms and shock (9, 11). These two patients were considered to have retropharyngeal abscesses and were treated with intravenous immunoglobulin and steroids after the appearance of cardiogenic shock. This case, along with the two previously reported MIS-C cases, suggests that early administration of intravenous immunoglobulin might prevent the progression of MIS-C and the development of circulatory failure. To verify our hypothesis, it is necessary to confirm in numerous cases that early administration of IVIG may prevent the exacerbation of severe conditions such as cardiogenic shock in MIS-C.suspected It is important to distinguish between retropharyngeal edema and retropharyngeal abscess for early MIS-C intervention. In addition to performing contrast-enhanced CT to confirm retropharyngeal edema, febrile children with severe neck involvement should be checked for a recent history of COVID-19 if MIS-C is suspected. Furthermore, since some of the clinical manifestations of MIS-C and Kawasaki disease overlap, biomarkers to differentiate between MIS-C and Kawasaki disease have been investigated (15). A reliable biomarker that predicts MIS-C will be useful for early intervention and improve the prognosis of patients with MIS-C.

## Conclusions

We described a previously healthy Japanese boy with suspected MIS-C–associated retropharyngeal edema. Although he did not improve with intensive antibiotic therapy, he recovered quickly after intravenous immunoglobulin treatment and did not develop severe clinical manifestations such as cardiac shock. Therefore, retropharyngeal edema might allow for the prediction of MIS-C and early intervention might prevent disease progression.

## Data Availability

The original contributions presented in the study are included in the article, further inquiries can be directed to the corresponding author.

## References

[1] Dufort EM Koumans EH Chow EJ Rosenthal EM Muse A Rowlands J Multisystem inflammatory syndrome in children in New York state. N Engl J Med. (2020) 383(4):347–58. 10.1056/NEJMoa2021756 32598830 PMC7346766

[2] Kaushik S Aydin SI Derespina KR Bansal PB Kowalsky S Trachtman R Multisystem inflammatory syndrome in children associated with severe acute respiratory syndrome coronavirus 2 infection (MIS-C): a multi-institutional study from New York city. J Pediatr. (2020) 224:24–9. 10.1016/j.jpeds.2020.06.045 32553861 PMC7293760

[3] Henderson LA Canna SW Friedman KG Gorelik M Lapidus SK Bassiri H American College of rheumatology clinical guidance for multisystem inflammatory syndrome in children associated with SARS-CoV-2 and hyperinflammation in pediatric COVID-19: version 2. Arthritis Rheumatol. (2021) 73(4):e13–29. 10.1002/art.41616 33277976 PMC8559788

[4] https://emergency.cdc.gov/han/2020/han00432.asp.

[5] https://www.who.int/news-room/commentaries/detail/multisystem-inflammatory-syndrome-in-children-and-adolescents-with-covid-19.

[6] Uehara R Belay ED. Epidemiology of kawasaki disease in Asia, Europe, and the United States. J Epidemiol. (2012) 22(2):79–85. 10.2188/jea.JE20110131 22307434 PMC3798585

[7] Wessels PA Bingler MA. A comparison of kawasaki disease and multisystem inflammatory syndrome in children. Prog Pediatr Cardiol. (2022) 65:101516. 10.1016/j.ppedcard.2022.101516 35313700 PMC8925196

[8] Tomomori A Hashida Y Sakata S. Five cases of kawasaki disease requiring differentiation from posterior pharyngeal abscess. Kawasaki disease requiring differentiation from posterior pharyngeal abscess. Syounika Rinshyo. (2014) 67:2159–64. in Japanese.

[9] Zhou C Cheng M Hong H. A mysterious fever and retropharyngeal edema on a previously healthy 10-year-old boy without known exposure to COVID-19. Cureus. (2022) 14(5):e25373. 10.7759/cureus.25373 35765385 PMC9233621

[10] Brooks R Fisher R Glicksman C Pollak U Simanovsky N Berkun Y. Multisystem inflammatory syndrome in children associated with COVID-19 presenting as cervical inflammation. Acta Paediatr. (2023) 112:477–82. 10.1111/apa.16623 36495064 PMC9877739

[11] Han P Douillard J Cheng J Ramanathan A Tieu D Degner T. Multisystem inflammatory syndrome in children in a 15-year-old male with a retropharyngeal phlegmon. Case Rep Pediatr. (2020) 2020:6668371. 10.1155/2020/6668371 33274096 PMC7678743

[12] Gupta P Giri PP Das D Pal P. Pediatric inflammatory multisystem syndrome (PIMS) presenting with retropharyngeal phlegmon mimicking kawasaki disease. Clin Rheumatol. (2021) 40(5):2097–8. 10.1007/s10067-020-05538-x 33439384 PMC7804211

[13] Jenkins E Sherry W Smith AGC Rostad BS Rostad CA Jones K Retropharyngeal edema and neck pain in multisystem inflammatory syndrome in children (MIS-c). J Pediatric Infect Dis Soc. (2021) 10(9):922–5. 10.1093/jpids/piab050 34173667 PMC8557366

[14] Daube A Rickert S Madan RP Kahn P Rispoli J Dapul H. Multisystem inflammatory syndrome in children (MIS-C) and retropharyngeal edema: a case series. Int J Pediatr Otorhinolaryngol. (2021) 144:110667. 10.1016/j.ijporl.2021.110667 33752089 PMC7931672

[15] Bukulmez H. Current understanding of multisystem inflammatory syndrome (MIS-C) following COVID-19 and its distinction from kawasaki disease. Curr Rheumatol Rep. (2021) 23(8):58. 10.1007/s11926-021-01028-4. 34216296 PMC8254432

